# The effects of barbed suture on watertightness after knee arthrotomy closure: a cadaveric study

**DOI:** 10.1186/s13018-018-1035-3

**Published:** 2018-12-20

**Authors:** Shu Kobayashi, Yasuo Niki, Kengo Harato, Kazuhiko Udagawa, Morio Matsumoto, Masaya Nakamura

**Affiliations:** Department of Orthopaedic Surgery, School of Medicine, Keio University, 35 Shinanomachi, Shinjuku, Tokyo 160-8582 Japan

**Keywords:** Barbed suture, Total knee arthroplasty, Watertightness, Arthrotomy closure

## Abstract

**Background:**

Wound closure is one of the crucial aspects of total knee arthroplasty (TKA) for patients who perform high-flexion activities of daily living, because the joint capsule is highly stretched and integrity of the arthrotomy closure must be maintained. Watertightness of the knee joint is a different aspect of the repair integrity of arthrotomy closure and is being noticed with increasing usage of the drain clamp method for blood management after TKA. Recently, the barbed knotless suture has been growing in popularity as a strong, secure closure appropriate for high-tension areas, such as the fascia and joint capsule. The purpose of this study was to compare the barbed knotless suture with simple interrupted suture in cadaveric knees.

**Methods:**

Nine fresh-frozen cadaveric lower extremities were used. After placing a parapatellar incision and setting a closed suction drain, arthrotomies were closed randomly using three suture materials: simple interrupted absorbable suture (No. 0 PDS, group C); or a single running knotless barbed suture Stratafix with 8N (group BS-8N) or 15N (group BS-15N) of tension. After arthrotomy closure, saline was injected in a retrograde manner into the joint through a drain until saline started to leak from the joint. Peak values for intra-articular pressure and infusion volume in each group were recorded and compared.

**Results:**

Mean infusion volumes were 13.0 ± 7.2 ml, 38.6 ± 10.7 ml, and 5.1 ± 2.5 ml in groups BS-8N, BS-15N, and C, respectively. Mean intra-articular pressures were 0.67 ± 0.47 kPa, 9.44 ± 4.55 kPa, and 0.56 ± 0.44 kPa in groups BS-8N, BS-15N, and C, respectively. Infusion volume and joint internal pressure were significantly higher in group BS-15N than in groups BS-8N (*p* = 0.008) or C (*p* = 0.04).

**Conclusions:**

Barbed suture with 15N appears appropriate for maintaining maximal watertightness after knee joint capsule closure, offering successful drain clamping, higher resistance to early mobilization protocols, and subsequent achievement of early deep knee flexion after TKA.

**Electronic supplementary material:**

The online version of this article (10.1186/s13018-018-1035-3) contains supplementary material, which is available to authorized users.

## Introduction

Despite continued improvements in surgical techniques and the management of pain and blood loss in total knee arthroplasty (TKA), about 20% of patients are dissatisfied with the clinical results [1]. One of the factors contributing to dissatisfaction is impairment of deep knee flexion, especially for Asian populations in which individuals are often engaged in activities of daily living that require deep knee flexion. Considering early mobilization protocols for deep knee flexion, both the arthrotomy closure method and the suture materials are crucial. Traditionally, arthrotomy has been closed by simple interrupted suture with multiple knots, which is time-consuming and creates uneven tension. From this perspective, the barbed suture allows simultaneous bidirectional knotless suturing, saving time and distributing tension equally along the entire length of the incision.

Due to the advantage of reduced blood loss, a drain clamp method with retrograde injection of tranexamic acid has been widely used in state-of-the-art TKA [2, 3]. Minimization of fluid leakage from the operative wound and maintenance of watertightness are key to successful drain clamping. We hypothesized that the barbed suture would prove beneficial for maintaining the watertightness of the drain clamp method and subsequently reduce blood loss. The purpose of this study was to compare peak intra-articular internal pressure and infusion volume during arthrotomy closure between the barbed suture and simple interrupted suture in cadaveric knees.

## Materials and methods

**Fig. 1:**
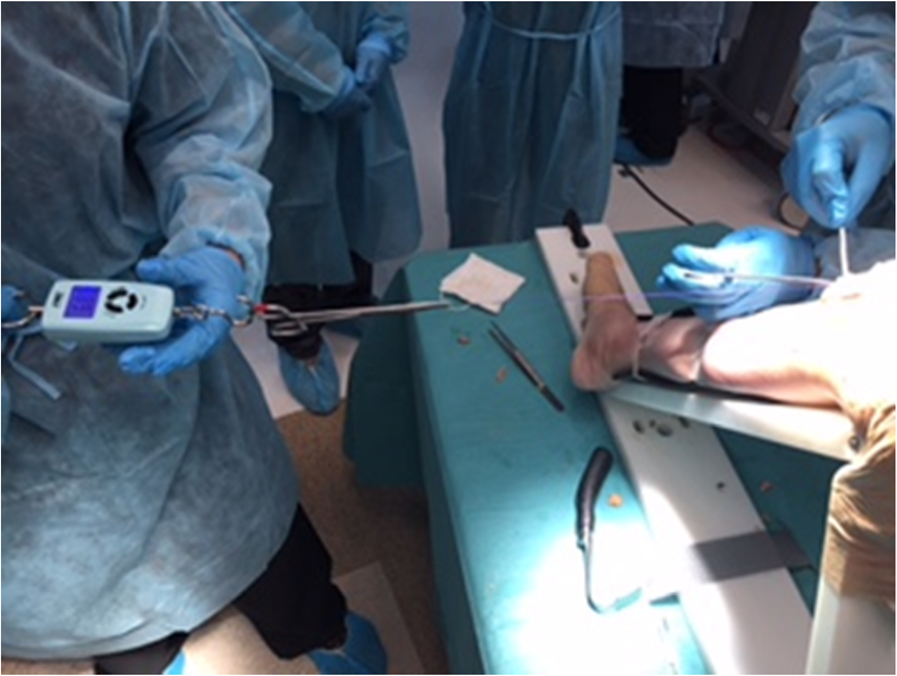
Each throw in both barbed suture groups was checked for tension using a spring balance

**Fig. 2:**
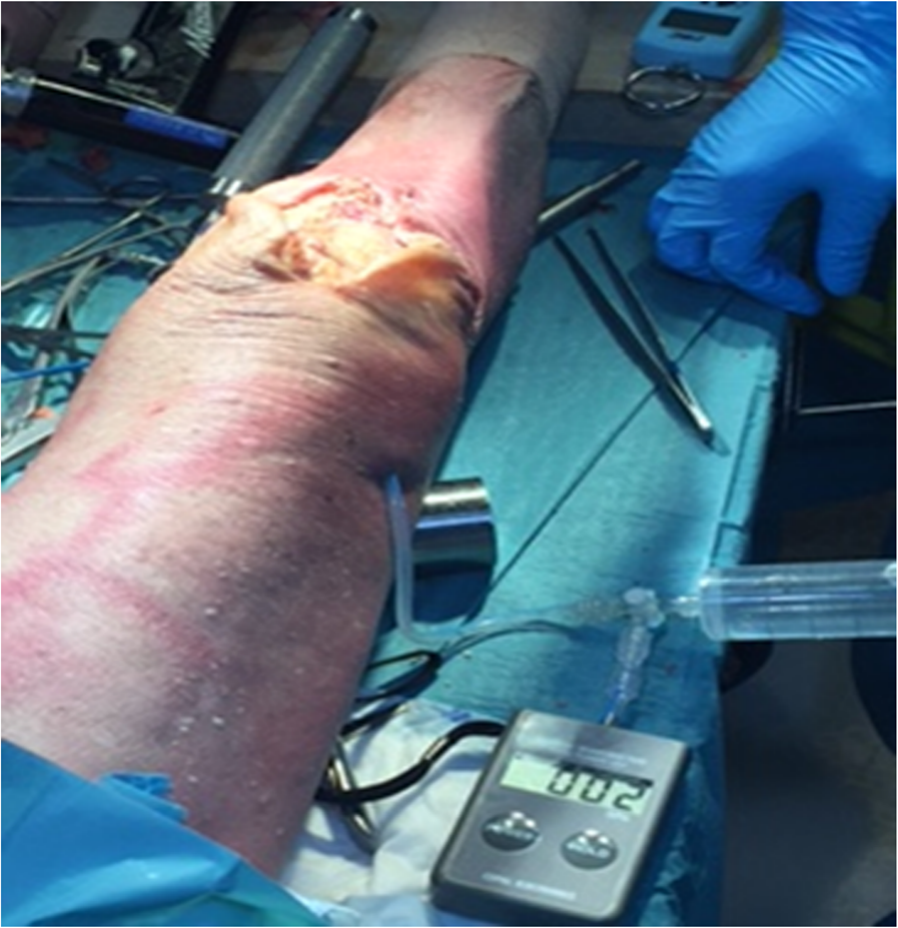
After capsule closure, saline was injected in a retrograde manner into the knee joint through a drain, which was connected to a manometer, until saline leaked from the joint

Nine fresh-frozen cadaveric lower extremities were used. After generous subcutaneous exposure, a standard medial parapatellar arthrotomy was performed from 4 cm proximal to the superior pole of the patella to 4 cm distal to the inferior pole of the patella on each knee. Total arthrotomy length was about 12 cm. After opening the knee joint with a parapatellar approach, a drain was fixed in the joint. The drain was then connected to a manometer (Handheld Digital Manometer; Nidec Copal Electronics, Tokyo, Japan) with a three-way stopcock (Terufusion®; Terumo, Tokyo, Japan). After that, arthrotomies were closed with the knee in approximately 30° of flexion. Knees were randomly assigned to three groups: group C, conventional interrupted absorbable sutures using No. 0 polydioxanone (PDS, Somerville, NJ, USA), and single running knotless bidirectional barbed absorbable suture (Stratafix®; Ethicon, Somerville, NJ, USA) with tension of 8N and 15N, designated as group BS-8N and group BS-15N, respectively. Each throw in both BS groups was checked for tension using a spring balance (Electrical scale; TenKou Syoji, Osaka, Japan) (Fig. 1). In all groups, each throw or suture was inserted into the full thickness of the tissue 10 mm on either side of the edge of the arthrotomy, and each throw or stitch was placed 20 mm apart. After capsule closure, saline was injected in a retrograde manner into the knee joint through a drain, until saline leaked from the joint (Fig. 2). At the time of leakage, peak intra-articular pressure was measured with a manometer and total infusion volume was recorded. All measurements were repeated three times and the three conditions of groups BS-8N, BS-15N, and C were tested in each knee. These data were collected from each of the nine cadavers. Mean values were then compared between the three groups.

### Statistical analysis

One-factor repeated-measures analysis of variance was performed. This was considered an appropriate statistical analysis for our study, in which the same parameters (i.e., infusion volume or intra-articular pressure) were measured under three different conditions within the same cadaveric knee.

## Results

**Fig. 3:**
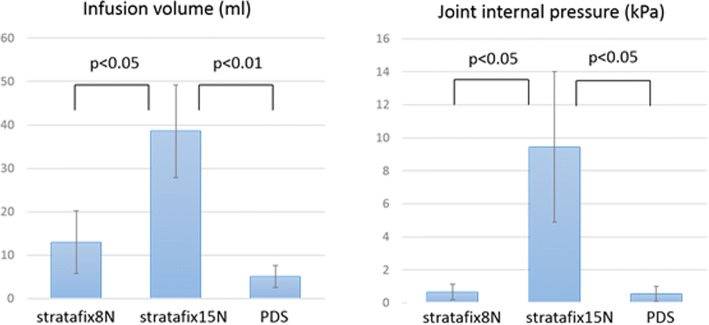
Infusion volume (*p* = 0.008) and joint internal pressure (*p* = 0.04) were significantly higher in group St15N than in group St8N or group C

In all groups, arthrotomy closure survived without rupture after 10 cycles of 140° knee flexion. Mean infusion volumes were 13.0 ± 7.2 ml, 38.6 ± 10.7 ml, and 5.1 ± 2.5 ml in groups BS-8N, BS-15N, and C, respectively. Mean intra-articular pressures were 0.67 ± 0.47 kPa, 9.44 ± 4.55 kPa, and 0.56 ± 0.44 kPa in groups BS-8N, BS-15N, and C, respectively. Mean infusion volume was significantly higher in group BS-15N than in group BS-8N (*p* = 0.008). Mean intra-articular pressure was significantly higher in group BS-15N than in group BS-8N (*p* = 0.04) (Fig. 3).

## Discussion

The barbed suture is a relatively new suture technique that was first described in 2007 and is now widely used for both skin and deeper structures. Positive aspects of the suture material are the specifically monofilament design with barbs orientated in the direction opposite the needle. Currently, three types of barbed sutures are commercially available: the Quill (Angiotech Pharmaceuticals, Vancouver, Canada), a bidirectional barbed suture; the V-Loc (Covidien, Mansfield, MA, USA), a unidirectional barbed suture that has only one needle and a loop at the end; and the Stratafix® (Ethicon), as an antibacterial monofilament suture with multiple small anchors on the string surface. The suture thread is absorbable but maintains its tensile strength for 6 weeks, providing secure closure appropriate for high-tension areas such as the fascia and joint capsule.

Barbed sutures have now been applied in orthopedic surgeries, as in other specialties. Even in the field of TKA, several studies have favored barbed sutures, especially for joint capsule closure due to the following advantages. First, the barbed suture is self-anchoring, requiring no knots, thus allowing faster closure than conventional interrupted sutures. Recent studies have reported that use of a barbed suture saved 3–12 min in TKA compared with a conventional suture [4–9], suggesting operative time savings that could substantially reduce the overall cost of TKA. Actually, several studies have indicated that barbed closures were associated with cost savings of between USD 30 and USD 550 per procedure [5–7, 9, 10]. However, in reality, some countries cap the prices of suture materials and regulate the margins for manufacturers, so appropriate evaluations of cost-effectiveness are difficult. Second, the barbed suture was experimentally confirmed to hold tightly in place, even if one of the throws breaks halfway. If a running traditional suture fails, nothing prevents the entire closure from failing due to the smooth nature of the stitch [11]. In practical terms, an accelerated rehabilitation protocol that is more robust to the cyclical loading of deep knee flexion requires integrity of the arthrotomy closure. In Asian populations with lifestyles involving deep knee flexion > 145° [12, 13], an early mobilization protocol after TKA is implemented, so that the skin area around the patella is highly stretched, and a large amount of stress is applied to the wound in the early postoperative phase.

In addition to these reported advantages, the watertightness of the knee joint is becoming ever more important with the increasing usage of the drain clamp method for blood management after TKA. Arthrotomy leakage after drain clamping would potentially reduce the efficacy of intra-articular injection of tranexamic acid and increase subcutaneous hematoma and wound complications, so watertight arthrotomy closure is mandatory. Previous biomechanical studies in cadavers have shown that a barbed suture provides a more watertight arthrotomy closure [14, 15], supporting our findings that the peak levels of intra-articular pressure and infusion volume during capsule closure were significantly higher and larger than those with conventional suture. Furthermore, our results indicated that 15N of tension per throw, but not 8N, was appropriate for achieving watertight capsule closure. However, cadaveric knee joints in this study were fresh frozen, so the condition of soft tissue was substantially different from that in real knee joints. Clarification of practical tension during arthrotomy closure using barbed suture in real knee joints should be planned.

Finally, some limitations need to be considered with regard to this study. First, our experiments were performed using cadaveric knees with no TKA implantation. Actually, we also performed similar arthrotomy closure in the BS-15N group after TKA implantation in four cadaveric knees and compared peak intra-articular pressure and infusion volume between knees with and without prosthetic implantation. The results showed that intra-articular pressure did not increase in proportion to the infusion volume of saline in the TKA implantation group (Additional file 1: Figure S1). This is attributed to the fact that the joint capsule is frequently perforated during release of the medial or posterior capsule at the time of TKA. However, we believe that when the extra-articular leakage of injected tranexamic acid from the operative wound is prevented by the barbed suture, hidden blood loss through perforations might be successfully reduced. Second, of the three commercially available barbed sutures, only Stratafix® was examined in this study. Third, the number of fresh cadaveric specimens available was limited. Last, although data were collected from nine cadavers, with three different conditions (BS-8N, BS-15N, and C) tested successively in each knee, variations in age, weight, and knee size of cadaver specimens represented unavoidable sources of potential bias in this study. Despite these limitations, the results suggest that the barbed suture represents an effective suture procedure with high watertightness, allowing successful drain clamping, high resistance to early mobilization, and early achievement of deep knee flexion after TKA.

## Conclusion

Barbed suture at 15N was appropriate for maintaining high watertightness after knee arthrotomy closure, successful drain clamping, high resistance to early immobilization, and subsequent achievement of early deep knee flexion after TKA.

## Additional file

Additional file 1: **Figure S1.** Intra-articular pressure did not increase in proportion to the infusion volume of saline in the TKA implantation group. (TIF 39 kb)

## Abbreviation

TKA: Total knee arthroplasty
