# Supplementary figures and images for: The effects of barbed suture on watertightness after knee arthrotomy closure: a cadaveric study
Source: J Orthop Surg Res. 2018 Dec 20;13:323. doi: 10.1186/s13018-018-1035-3 (PMC6302503; doi:10.1186/s13018-018-1035-3)

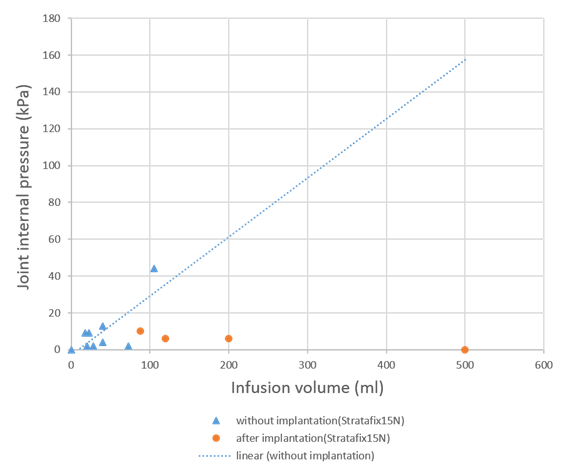

Supplement: Supplementary file 1 — Figure S1. Intra-articular pressure did not increase in proportion to the infusion volume of saline in the TKA implantation group. (TIF 39 kb) [file 13018_2018_1035_MOESM1_ESM.tif]
